# Maternal Seroprevalence and Placental Transfer of COVID-19 Antibodies in Pregnancy: A Hospital-Based Study

**DOI:** 10.7759/cureus.49730

**Published:** 2023-11-30

**Authors:** Joseph Okoeguale, Okelue E Okobi, Emmanuella C Ojukwu, Onyinyechukwu B Nwachukwu, Caroline C Okoroafor

**Affiliations:** 1 Obstetrics and Gynaecology, Irrua Specialist Teaching Hospital, Irrua, NGA; 2 Family Medicine, Larkin Community Hospital Palm Springs Campus, Miami, USA; 3 Family Medicine, Medficient Health Systems, Laurel, USA; 4 Family Medicine, Lakeside Medical Center, Belle Glade, USA; 5 Obstetrics and Gynecology, St. George's University School of Medicine, Brooklyn, USA; 6 Neurosciences and Psychology, California Institute of Behavioral Neurosciences & Psychology, Farfield, USA; 7 Family Medicine, American International School of Medicine, Georgetown, USA; 8 General Practice, University of Calabar, Calabar, NGA

**Keywords:** polymerase chain reaction, antibody transfer, covid-19, pregnancy, seroprevalence

## Abstract

Background: Coronavirus disease 2019 (COVID-19) is a relatively new disease with high morbidity and mortality. Information about the prevalence of infections in pregnancy could help identify herd immunity, project epidemics, and decide policy guidelines.

Objectives: The aim of this study was to determine the infection susceptibility risk of COVID-19 in pregnancy, to determine the prevalence of COVID-19 antibodies (IgG & IgM), and to evaluate the determinants of COVID-19 antibody positivity in pregnancy.

Materials and methods: This was an analytical cross-sectional study involving 258 consenting pregnant women recruited at Irrua Specialist Teaching Hospital, Edo State, Nigeria. Of these, 179 participants were recruited from the antenatal clinic, and 79 from the gynecology emergency unit. A structured questionnaire was administered at baseline. Venous blood was obtained at enrolment to test for total antibodies using ELISA. A nasopharyngeal swab was simultaneously obtained for COVID-19 PCR for all participants. Umbilical cord blood was taken after delivery in those who had positive serology. Socio-demographic variables and clinical presentation of respondents were considered as exposure variables, and this was cross-tabulated with outcome variables in bivariate analysis using chi-square with a level of significance at a P-value less than 0.05. Variables in bivariate analysis of chi-square that have a P-value less than 0.2 were entered into a logistic regression using multivariate logistic models.

Results: The study detected active COVID-19 infections among 7.4% (19/258) of the study participants. The study demonstrated a seroprevalence of COVID-19 antibodies in 62.4% (161/258) of the participants at recruitment and showed a strong correlation between working in the healthcare setting and living in an urban environment. Our study also reported 5.3% (8/152) of cord blood antibody positivity among study participants. The concentration of maternal immunoglobulin strongly and positively correlated with cord blood seropositivity.

Conclusion: Prevalence estimates are an underestimate of the actual proportion of pregnant women with prior COVID-19 exposure as observed in the study discrepancy of confirmed PCR infection and evidence of previous infection from serology. The study also highlighted a low efficiency of placental transfer of COVID-19 antibodies at birth among those who were seropositive at baseline and showed that maternal antibody levels play an important role in determining the efficiency of placenta transfer of COVID-19 antibodies in pregnancy.

## Introduction

COVID-19, a potentially severe acute respiratory infection, is caused by the severe acute respiratory syndrome coronavirus 2 (SARS-CoV-2) [1,2]. COVID-19 manifestation spans from symptomatic to asymptomatic, with a majority of the infections going undiagnosed and unreported [3]. Consequently, relying solely on clinically apparent cases significantly underestimates the true infection rates. The existence of asymptomatic or subclinical infections raises concerns due to their potential for propagating the infection, which poses significant challenges to public health efforts. Serologic tests detect COVID-19-specific antibodies, indicating previous exposure regardless of symptom severity [4,5]. Monitoring seropositivity in a population reveals the infection extent. In pregnancy, viral infections pose risks due to altered immune status, increasing vulnerability to adverse outcomes [6]. Viral infections such as influenza, Lassa fever, and Ebola have elevated mortality rates for pregnant women and fetuses [7-9].

Despite extensive COVID-19 research, significant knowledge gaps remain, especially regarding pregnancy, with limited studies in Africa. Unfortunately, pregnant women are often excluded from the research on vaccines and treatments for emerging infectious diseases due to concerns about fetal risks, despite the threats posed by COVID-19 to mothers and healthcare workers [10,11]. It is crucial to recognize pregnant women as a unique and vulnerable group deserving specific research attention for COVID-19 and other emerging diseases. Moreover, understanding its effects during pregnancy remains limited, with emerging evidence suggesting a substantial portion of pregnant women are asymptomatic [12]. While numerous COVID-19 vaccines are in development, lacking comprehensive epidemiological data hampers trial design, with gaps in understanding seroprevalence, infection rates, high-risk groups, disease presentations, and antibody kinetics. It is also approximated that 80% of infections are mild or asymptomatic [13]. Therefore, relying solely on confirmed cases may underestimate the true disease burden and infection fatality rate. Serological tests are, therefore, recommended for the detection of specific antibodies, as this will provide an accurate estimate of infections [14-20]. A recent New York City-based study conducted between March and April 2020 found that 36% of the suspected COVID-19 pregnant patients tested positive [21]. In sub-Saharan Africa, asymptomatic pregnant women are rarely tested, hindering disease burden assessment. Pregnant women's frequent interactions heighten risks for healthcare workers and the public, warranting their classification as a "special group" in COVID-19 interventions. Nigeria's testing is mainly for symptomatic patients, potentially underestimating the COVID-19 disease burden. Philadelphia research reported 1.4% PCR-confirmed COVID-19 cases in pregnant women, contrasting serological studies showing a higher 6.2% exposure rate [22-26]. A Nigerian household seroprevalence survey revealed higher infection rates than national data, particularly in asymptomatic cases [25]. This survey found COVID-19 antibodies in 23% (Lagos state) and 9% (Gombe state), indicating substantial exposure [25]. Seroprevalence surveys effectively estimate population exposure.

Serology testing is gaining importance in detecting COVID-19 exposure and transmission. Patients typically show positive IgG or IgM antibody results within 19 days of SARS-CoV-2 exposure, even after negative PCR results [27-31]. Pregnant women and neonates have unique immune systems shaped by maternal-fetal interactions. The extent of passive transfer of COVID-19 antibodies is underexplored, with cases of neonates born to mothers with differing serologic statuses raising uncertainties [32-36], including asymptomatic women's ability to transfer antibodies [36]. Presently, COVID-19 detection is reliant on PCR tests, which analyze respiratory samples for ongoing infections [37], and serology, which examines blood for past infections [37]. Thus, a positive PCR test confirms active COVID-19 presence [38], while a positive antibody test indicates prior infection [38]. Currently, the gold standard diagnosis is positive RT-PCR results from respiratory specimens [39,40]. Combining RT-PCR, clinical symptoms, and serological testing ensures timely and accurate diagnosis, especially in pregnant women. COVID-19 lacks sufficient pregnancy-related data. Serological tests are crucial for understanding viral exposure, especially in pregnant women, who consistently engage with healthcare workers during prenatal care and delivery, offering insights into community exposure. Therefore, there is a need to ascertain asymptomatic infections in pregnant women and the immunity proportion among previously infected individuals. Monitoring antibody seropositivity (IgG and IgM) can gauge infection extent and incidence, aligning with WHO guidelines. Further, it is acknowledged that pregnant women are increasingly vulnerable to viral infections, with attendant risks of severe maternal and fetal outcomes. As such, conducting the study on pregnant Nigerian women enables the evaluation of clinical and epidemiological patterns, alongside the COVID-19 and obstetric outcomes in pregnant women visiting Nigerian hospitals for prenatal care services.

This study’s objectives include the estimation of COVID-19 seropositivity during pregnancy and antibody transfer at delivery; the determination of the COVID-19 infection rates among symptomatic pregnant women presenting for antenatal care in the study area, as well as ascertaining the incidence of asymptomatic COVID-19 infections among pregnant women within the same region; establishing the prevalence of COVID-19 antibodies (IgG and IgM) in symptomatic and asymptomatic pregnant women; and the evaluation of the proportion of respondents with COVID-19 cord blood seropositivity, shedding light on the potential transmission of antibodies from seropositive mothers to their newborns.

## Materials and methods

Study area, study population and design, and sampling technique

This study was conducted at Irrua Specialist Teaching Hospital (ISTH) in Irrua, Edo State, Nigeria. ISTH, established in 1991, is one of Edo State's federal tertiary hospitals, serving as a referral center for the region. It receives patients from various healthcare institutions and plays a vital role in diagnosing and managing emerging infectious diseases such as COVID-19, Lassa fever, monkeypox, Marburg, and yellow fever. ISTH conducts reverse-transcription polymerase-chain-reaction (RT-PCR) testing and serves patients from all over Nigeria. Participants included pregnant women attending ISTH's antenatal clinic and seropositive mothers. This analytical cross-sectional study involved recruitment at two stages: baseline (enrollment) and delivery (cord blood sampling). A multi-stage sampling method was used for participant selection.

Selection criteria (inclusion and exclusion criteria)

Inclusion criteria included (a) pregnant women aged ≥18 years, attending the antenatal clinic or presenting at the Maternal and Child Health Unit (MCH) Casualty Unit at ISTH Irrua; (b) women who intend to deliver at Irrua Specialist Teaching Hospital at full term; and (c) pregnant women who test seropositive at recruitment and consent to cord blood sampling upon delivery. The exclusion criteria included (a) pregnant women below 18 years of age and (b) pregnant women who declined participation in the study. By adhering to these selection criteria, the study aims to gather meaningful insights into the susceptibility, seropositivity, and antibody transfer dynamics of COVID-19 among pregnant women in the specified region.

Outcome measures

The primary outcome measures of this study encompass various aspects: (a) the prevalence of COVID-19 infection in pregnant women, encompassing both symptomatic and asymptomatic cases; (b) the seroprevalence of COVID-19 antibodies within the study population; and (c) the COVID-19 seropositivity in cord blood among mothers who tested seropositive. The secondary outcome revolves around understanding the factors associated with COVID-19 seropositivity in pregnant women and cord blood.

Recruitment at baseline

Participants entered the study via the MCH, which serves a diverse group of pregnant women, including those receiving antenatal care, those with pregnancy-related complaints, and those in labor. Pregnant women accessed the unit through various routes, including the antenatal clinic (for routine care), the emergency unit (for complaints such as fever or respiratory symptoms), or the labor ward (for those in labor). Symptomatic patients occasionally came directly through their antenatal clinics. Weekly records showed the MCH attended to at least 222 antenatal care seekers and about 98 gynecological emergency cases. The antenatal clinic operated from Monday to Thursday, while the gynecological emergency unit was open all week.

Determination of the sample size, sampling frame, and proportionate random sampling per unit

Sample size calculations considered a 95% confidence level and a two-sided type 1 error of 0.05. The estimated seroprevalence of COVID-19 in Nigeria was 18.5% across four states. Using the formula N = Z ^ 2pq/d ^ 2, where N represents the minimum sample size, Z is the critical value (1.96 for 95% confidence), p is the prevalence (18.5%), q is the complementary probability (0.815), and d is the degree of accuracy (0.05). The initial sample size (N) was computed as 232 subjects. Accounting for a 10% non-response rate, the adjusted sample size (ns) became 256 participants.

Our sampling frame encompassed patients accessing the antenatal clinic and gynecological emergency unit, totaling 320 women weekly. Proportionate sampling over four weeks projected 888 women from the antenatal clinic and 392 from the gynecological emergency unit. Combining both units, 1,280 women were expected over four weeks, and the study's sample size was set at 258 participants.

For the gynecological emergency unit, the proportionate allocation was calculated by dividing its projected participants by the total projected for both units and then multiplying by the study's sample size, resulting in 79 participants. Similarly, for the antenatal clinic, proportionate allocation yielded 179 participants. In total, 179 participants were recruited from the antenatal clinic, and 79 from the gynecological emergency unit.

Systematic sampling for recruitment of study participants

The sampling interval (nth number) was determined by taking a percentage of the desired participants to be seen over the four-week period. For the gynecological emergency unit, every 20th patient presenting to the unit was selected (79/392 X 100 = 20th patient), and the same was done for the antenatal clinic (179/888 X 100 = 20th patient). The first participant was selected through simple random sampling via balloting. The initial 20 patients received a bag containing 20 tallies, with 19 labeled "No" and one labeled "Yes." In our study, the sixth patient was the first to be recruited, followed by every 20th patient thereafter. When a selected participant did not meet the eligibility criteria, was absent during data collection, or opted out, the next eligible participant was recruited. Trained healthcare staff, including doctors and nurses at ISTH's Maternal and Child Health Unit, also identified eligible participants during their visits and consultations at the unit.

Study visits

This study was conducted between September 2021 and July 2022. During enrollment, a structured questionnaire collected baseline data, including socio-demographics (age, gender, parity, education, risk exposure history), and recorded presenting symptoms such as fever, cough, and loss of smell. COVID-19 PCR and serology tests were performed, along with cord blood collection for serology. Gestational age was determined using ultrasound and the last menstrual period. For seropositive mothers, newborns' cord blood was sampled at birth.

Questionnaires were administered face-to-face in English, with translated versions for non-English speakers. All participants received standard antenatal and post-natal care per hospital protocols. Symptomatic participants exhibited acute illness with symptoms such as fever or respiratory distress, while asymptomatic participants had routine antenatal visits without complaints.

Sample collection, processing, and storage

Upon enrollment, 7.5 mL of venous blood was collected for total antibody assessment via ELISA, while a nasopharyngeal swab was taken for COVID-19 RT-PCR. Immunoglobulin-positive individuals had 7.5 mL of umbilical cord blood collected post-delivery, stored in serum separator tubes, and processed in a dedicated molecular laboratory.

Questionnaires were completed in a consulting room, with forms and containers labeled with unique identification, date, and time. Full personal protective equipment (PPE), including aprons, gloves, face shields, and N95 masks, was worn throughout. Translation into the local language was provided as needed.

COVID-19 testing procedures

The nasopharyngeal swab was inserted into the nostril for 20 seconds to absorb secretions and then placed in sterile tubes containing viral transport media (VTM). The VTM tube was securely wrapped in absorbent material and inserted into a leak-proof secondary container, which was further enclosed in a centrifuge or falcon tube and a zip-lock bag labeled with a biohazard sign. COVID-19 samples were transported with ice-cold packs. RealStar® SARS-CoV-2 RT-PCR Kit RUO (Altona Diagnostics, Hamburg, Germany) was used for COVID-19 PCR, while total antibody levels were assessed using the Wantai SARS-CoV-2 Ab ELISA diagnostics (Wantai BioPharm, Beijing, China).

Data analysis

Data analysis was conducted using Statistical Product and Service Solutions (SPSS, version 25.0) (IBM SPSS Statistics for Windows, Armonk, NY). The analysis involved descriptive and bivariate analyses, along with multivariate analysis using logistic regression models. Initially, a descriptive analysis was performed to understand the key characteristics of participating pregnant women. Summary statistics, including mean, standard deviation, median, interquartile range, range, frequency, and proportions, were used as appropriate measures.

In the analytical process, socio-demographic and clinical presentation variables were considered exposure variables. These variables were cross-tabulated with outcome variables (seropositive and seronegative respondents) in a bivariate analysis using the chi-square test, with a significance level set at P < 0.05. Variables with a P-value less than 0.2 in the bivariate chi-square analysis were selected for inclusion in the multivariate logistic regression model.

## Results

Study participants and characteristics

A total of 258 study participants were enrolled in the investigation, with 179 originating from the antenatal clinic and 79 from the gynecology emergency unit. Among these participants, 152 pregnant women presented without any symptoms, while 106 pregnant women exhibited symptoms at the time of recruitment. The average age of the study participants was 30.6 ± 4.0 years, with an age range spanning from 18 to 44 years. Pregnant women aged between 26 and 30 years accounted for 37.0% of the entire study cohort.

Furthermore, a substantial portion of the participants (69.8%) had experienced at least one previous pregnancy, with the majority currently in either their second or third trimester. Education levels were notable among the participants, as 54.7% had successfully completed secondary school. In terms of residence, 68.2% of the participants hailed from rural areas. Comprehensive information detailing the baseline characteristics of the study participants is available in Table 1 below. These characteristics serve as essential context for the subsequent analyses and findings of the study.

**Table 1:** Baseline characteristics of pregnant women Others: House-help, cosmetic shop worker, security guard, casual laborers, contract workers in hotels and restaurants. HIV = human immunodeficiency virus, IQR = interquartile range; n = number of pregnant women recruited into the study; SD = standard deviation

Characteristics	N	%
Total sample size	258	100
Age (years)		
≤ 25	53	20.5
26-30	95	36.8
31-35	69	26.7
36-40	41	15.9
Mean age ± SD [range]	30·6 ± 4·01 [18 – 44]	-
Gestational age (weeks)		
≤ 13	8	3.1
14-27	116	45
>28	134	52
Median [IQR]	25 [08 - 36]	-
Area of residence		
Lives in a rural area	176	68.2
Lives in Urban Area	82	31.5
Educational level		
No formal education	6	2.3
Primary	58	22.5
Secondary	141	54.7
Tertiary	53	20.5
Occupation		
Trading	126	48.9
Civil/public service	50	19.4
Health Care Workers	28	10.9
Farming	20	7.8
Housewife	12	4.7
Artisan	9	3.5
Others	13	5
Parity		
0	46	17.8
1-4	180	69.8
≥ 5	32	12.4
Total number of symptomatic pregnant women recruited	106	41.1
Total number of asymptomatic pregnant women recruited	152	58.9
HIV	7	2.7
Hepatitis B infected participants	2	0.8

COVID-19 positivity rate among study participants (n=258)

**Figure 1:**
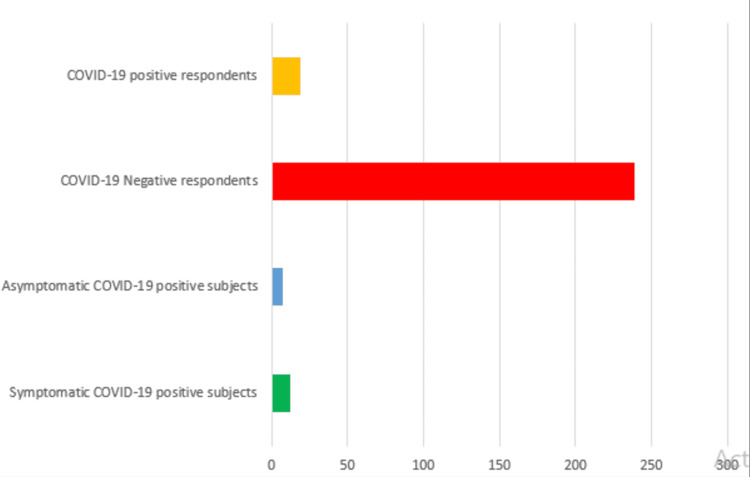
COVID-19 rates among the study participants

The analysis revealed that the incidence of COVID-19 RT-PCR positivity among the study participants was calculated to be 7.4%, with 19 out of 258 participants testing positive for the virus. Among these 19 positive cases, it was observed that seven individuals (36.8%) were asymptomatic at the time of testing, whereas 12 participants (63.2%) presented with symptoms indicative of COVID-19 during their recruitment into the study. This breakdown (see Figure 1 below) highlights the varying clinical presentations of COVID-19 within the cohort of participants under investigation.

Of the overall study size of 258, 152 study participants did not present with symptoms (see Table 2 below), the incidence of coronavirus disease was 4.6%, and about 95.4% was negative for RT-PCR.

**Table 2:** Result of asymptomatic respondents

Asymptomatic respondents (n=152)	Sum of frequency	Sum of percentage
COVID-19 Positive	7	4.6
COVID-19 Negative	145	95.4
Grand Total	152	100

Among the study participants who presented with coronavirus-related symptoms (see Tables 3-4 below), 106 of them were ill, presenting to the antenatal or emergency unit with these symptoms, 12 tested positive, and 94 negative to RT-PCR. The prevalence of coronavirus disease infection among symptomatic pregnant women recruited in the maternal and child health unit was 11.3%, and about 88.7% were negative using real-time PCR.

**Table 3:** Study participants who presented with coronavirus-related symptoms PCR = Polymerase chain reaction

Variables	Sum of frequency	Sum of percentage
COVID-19 PCR Negative	94	88.7
COVID-19 PCR Positive	12	11.3
Grand Total	106	100

**Table 4:** Common presenting clinical symptoms among the study participants N = number of symptomatic participants, n = number of symptomatic participants with confirmed coronavirus disease, PCR = polymerase chain reaction

Symptoms	N=106	Proportion of COVID-19 PCR Positives in symptomatic patients n =12
Cough	88	10 (83.3)
Fever	72	8 (66.7)
Fatigue	71	8 (66.7)
Chest pain	57	9 (75.0)
Diarrhea	22	4 (33.3)
Loss of smell	11	6 (50.0)
Difficulty with breathing	09	2 (16.7)
Visual blurring	04	1 (8.3)

Seroprevalence of COVID-19 among the study participants

The overall seroprevalence at enrolment of participants over the study period was 164/258 (63.57%), and 10.6% (17/161) of seropositive participants had active COVID-19 infection and reported COVID-19 PCR positive at the time of recruitment. The mean age among seropositive mothers was 29.8 ± 5.2 years (range: 18-40) and was not significantly different from that of seronegative mothers (P=0.435). In a multivariable logistic regression, working in a healthcare setting (aOR=6.01 (95%CI=1.83-44.8), P=0.02) and living in an urban environment (95%CI=2.66 (1.00-4.79), P=0.046) were significantly associated with COVID-19 immunoglobulin seropositivity (Table 5).

**Table 5:** Prevalence of COVID-19 antibodies

Total COVID-19 antibodies for symptomatic patients	Frequency (n=258)	Percent (%)
Positive	37	34.9
Negative	69	65.1
Total COVID-19 antibodies for asymptomatic patients	Frequency (n=152)	Percent (%)
Positive	109	71.7
Negative	45	29.6

Prevalence of COVID-19 antibodies in cord blood

Table 6 below indicates the overall seroprevalence of COVID-19 infection at enrollment of participants during the study period, including participants with positive and active PCR COVID-19 infection.

**Table 6:** Prevalence of COVID-19 antibodies in cord blood

Prevalence of COVID-19 antibodies in cord blood	Frequency (n=152)	Percent (%)
Cord blood COVID-19 seropositivity		
Positive	8	5.3
Negative	144	94.7

Transplacental transfer

Our study demonstrated a transplacental transfer of COVID-19 antibodies of 5.3% among seropositive mothers in the third trimester. Nine of the seropositive women were missing (lost to follow-up at delivery). There were variable factors associated with this seropositivity (see Table 7 below).

**Table 7:** Factors associated with COVID-19 immunoglobulin seropositivity For statistical significance, P<0.05 CI = confidence interval; GA = gestational age; n = number of seropositive women; N = total number of women who were enrolled in the study (258); OR = odds ratio; n = number of seropositive participants, (-): intentionally left blank

Variables	N=number of respondents	n=number of seropositive respondents	95% CI	P-value crude	OR adjusted	P-value adjusted
Age	≤25 = 53	30	1.01 (0.98-1.08)	0.246	-	-
	26-30 = 95	68	1·46 (0.24-9.3)	0.643	-	-
	31-35 = 69	39	0.83 (0.34-6.2)	0.341	-	-
	36-40 = 41	24	0.53 (0.16-1.58)	0.283	-	-
Gestational age	≤ 13 = 8	5	0.97 (0.95-1.02)	0.26	-	-
	14-27 = 116	71	1.24 (1.14-2.91)	0.425	-	-
	> 28 = 134	85	1.62 (0.96-3.42)	0.262	-	-
Parity	0 = 46	28	1.62 (1.06-4.81)	0.443	1.12 (0.42-8.76)	0.441
	1-4 = 180	121	1.15 (1.01-1.32)	0.143	-	-
	≥ 5 = 32	12	-	0.336	-	-
Self-reported prior history of treatment for COVID-19	No = 216	155	Reference 1.83 (0.84-4.7)	0.132	1.52 (0.90-2.55)	0.103
	Yes = 42	6	-	-	-	-
Healthcare worker	28	17	6.01 (1.83-44.8)	0.002	2·32 (1.18-4.60)	0.016
Area of residence	Urban = 82	67	2.66 (1.00-4.79)	0.043	3.21 (1.02-5.80)	0.047
	Rural = 176	94	-	-	-	-
Viral illness in pregnancy (HIV and/or HBsAg)	No = 249	159	0.53 (0.16-1.58)	0.267	-	-
	Yes = 09	2	-	-	-	-
Self-reported history of treatment for febrile illness	No = 214	132	1.60 (0.83-3.20)	0.374	-	-
	Yes = 44	29	-	-	-	-

Analysis of the effect of different factors on the placenta transfer of COVID-19 antibodies (cord blood seropositivity)

Our study reported 5.3% (8/152) of cord blood antibody positivity among study participants (see Table 8 below). The concentration of maternal immunoglobulin strongly and positively correlated with cord blood seropositivity. Nine of the participants who were seropositive at baseline were missing at delivery (lost to follow-up).

**Table 8:** Factor associated with cord blood antibody positivity among the study participants For statistical significance, P<0.05. CI = confidence interval; OR = odds ratio; n = number of cord blood antibody positive participants; HIV = human immunodeficiency viruses; HBsAg = hepatitis B surface antigen; (-): "intentionally left blank"

Variables	N = 8	[95% CI] crude	P-value crude	[95% CI] adjusted	[95% CI] adjusted
Positive history of COVID-19 infection	No = 7	Reference 0.012 (-0.62 to -0.65)	0.968	-	-
	Yes = 1	-	-	-	-
Maternal immunoglobulin concentration (reference index)	High = 8	Reference -0.12 (-0.19 to -0.02)	0.009	-0.112 (-0.19 to -0.03)	0.007
Gestational age at birth	≥ 37 weeks = 6	Reference 0.017 (-0.39 to 0.43)	0.976	-	-
	< 37 weeks = 2	-	-	-	-
Viral illness in pregnancy (HIV and/or HBsAg)	No = 8	Reference 0.074 (-0.45 to 0.59)	0.782	-	-
Sex at birth	Male = 3	Reference 0.081 (-0.12 to 0.29)	0.451	-	-
	Female = 5	-	-	-	-

## Discussion

COVID-19 seroprevalence among pregnant women

The relentless spread of coronavirus disease across Nigeria has impacted various demographics, including pregnant women, adults, and children, during multiple waves of the pandemic. To accurately gauge the infection's reach and grasp its true burden, it becomes imperative to establish a dependable estimate of COVID-19-specific antibodies. Vulnerabilities of pregnant women and children to viral infections underscore the necessity of their inclusion in studies focusing on emerging and re-emerging infectious diseases.

Our investigation revealed a noteworthy seroprevalence of COVID-19 immunoglobulins (total IgG and IgM) at 62.4% among pregnant women attending antenatal care at Irrua Specialist Teaching Hospital in Edo State, Nigeria. This finding suggests a susceptibility risk of 37.6% within the studied pregnant population. Interestingly, this risk contrasts significantly with the susceptibility risk of 78% reported in the general West African population and an alarming 94% among pregnant women in a meta-analysis of African COVID-19 seroprevalence studies published in 2022 [41]. Notably, the aforementioned study acknowledged limitations due to the scarcity of available data.

Our results align with the conclusions drawn by Price et al. [42] from a study conducted in Haiti, where a seroprevalence of 56.7% was reported among a group of pregnant hospital patients. It is worth noting that our findings contribute to the mounting evidence suggesting that the actual number of COVID-19 cases in Nigeria may surpass the officially reported figures, both within the West African region and certain parts of Europe [43].

In Nigeria, lax adherence to social distancing guidelines and mask-wearing protocols has been evident [41-44]. Overcrowded urban marketplaces and public transportation, coupled with limited alternatives, may have fostered a situation where a significant portion of the population, including pregnant women, was exposed to symptomatic and asymptomatic COVID-19 infections. In many instances, these cases might have been resolved through over-the-counter supportive treatments, a common scenario in Nigeria. While inadequate infection control measures could have contributed to the surge in COVID-19 seropositivity among pregnant individuals, the extent to which this exposure translates to the development of protective herd immunity remains uncertain and warrants further exploration.

Understanding COVID-19 incidence and seroprevalence

Following the successive waves of the COVID-19 pandemic, various regions in Nigeria have witnessed outbreaks of respiratory symptoms and febrile illnesses. However, due to limited testing resources, uncomfortable testing methods, and social stigma surrounding testing, these outbreaks remained unconfirmed as COVID-19 cases. This points to a significant underestimation of the actual COVID-19 numbers, potentially by several orders of magnitude. In our study, we observed a COVID-19 incidence rate of 7.4%, with rates of 4.6% among asymptomatic individuals and 11.3% among pregnant patients with febrile or respiratory symptoms. These figures stand in stark contrast to the peak periods of each pandemic wave, during which over 20-50% of patients presenting with respiratory symptoms tested positive for COVID-19 in our institution [44]. It is plausible that prior exposures may have conferred some level of protection against severe clinical manifestations, as indicated by the notable proportion of pregnant women with positive antibody results. However, this hypothesis requires thorough investigation.

Our findings did not indicate a significant association between age, gestational age, parity, and antibody seropositivity among respondents. Of particular interest is the lack of a significant correlation in seropositivity between study participants who reported treating COVID-19 during pregnancy and those managed for febrile illness. Nevertheless, a significant correlation was observed between urban dwellings and COVID-19 immunoglobulin seropositivity. Moreover, working within a healthcare setting demonstrated a significant association with COVID-19 seropositivity. Strikingly, population-based studies in Greece [45] and Spain [46] found higher seroprevalence rates in relation to age and urban living, even though overall COVID-19 antibody seropositivity remained low. This difference can be attributed to the elevated exposure risk to infectious diseases within healthcare environments, as reaffirmed by this study's findings, consistent with previous reports [47,48].

We also examined umbilical cord immunoglobulin levels among 5.3% of the 152 respondents sampled at delivery. A notable correlation between increasing maternal antibody concentration and cord blood COVID-19 seropositivity was observed. Interestingly, only eight respondents displayed placental transfer of immunoglobulin antibodies, as demonstrated by umbilical cord blood testing. It is worth noting that immunologic memory of COVID-19-specific antibodies, CD4+ T cells, CD8+ T cells, and memory B-cells, lasts between five to eight months based on cell lineage. However, this study's design lacked seroconversion studies, which would have necessitated repeat maternal blood sampling at delivery for those who tested positive at baseline. Such studies would have been vital in determining seroconversion status, representing a notable limitation in our research. Factors such as maternal conditions or infections, such as HIV infection and placental malaria, are known to influence antibody transfer efficiency [49-51], particularly given the malaria endemicity in many parts of Nigeria. A future approach could involve incorporating malaria rapid diagnostic tests or microscopy to identify malaria parasites in the sampled blood. Furthermore, neither HIV nor hepatitis B infections correlated positively with maternal or cord blood COVID-19 seropositivity, highlighting a complex interplay of factors in this context.

Strengths and limitation of this study

Among the notable strengths of this study includes the observation that it employed a structured questionnaire to gather comprehensive baseline data from 258 pregnant women, facilitating diverse participant inclusion and robust assessment of demographics and clinical characteristics. Employing both RT-PCR and serology tests enhanced disease prevalence understanding by identifying active infections and prior exposure, presenting a holistic view of COVID-19 within the cohort. The cross-sectional study design, complemented by logistic regression, explored factors affecting COVID-19 antibody seropositivity and placental transfer, enhancing association identification and confounding control. Focusing on pregnant women addresses a crucial research gap, providing insights into COVID-19's unique impact on this vulnerable group. Including symptomatic and asymptomatic participants and cord blood analysis adds depth to the findings, informing maternal-fetal health considerations. Leveraging real-world hospital data enhances external validity, allowing findings to be relevant to similar healthcare settings [51].

Nonetheless, these limitations warrant consideration: The sample size might not fully represent diverse populations. The participant composition introduces selection bias, limiting generalizability. The cross-sectional design prevents causal inference, warranting longitudinal approaches. Self-reported data raise recall bias possibilities. Lost follow-up participants and test inaccuracies introduce potential bias. Uncontrolled confounding variables might influence associations. The single-center setting and lack of follow-up visits limit broad context understanding. External factors such as public health measures and variants were unaccounted for. In conclusion, the study's strengths provide valuable insights, yet its limitations call for cautious interpretation and application in broader contexts and settings.

## Conclusions

This study serves to gauge the prevalence of COVID-19 infection within the pregnant population attending the Maternal and Child Health Unit of Irrua Specialist Teaching Hospital in Edo State, Nigeria. Our findings shed light on a crucial matter: prevalence estimates appear to underreport the true proportion of pregnant women who have previously encountered COVID-19. This incongruence is vividly exemplified by the stark contrast between confirmed PCR infection cases and the indications of past infection from serological evidence. Furthermore, a significant insight emerged concerning the efficiency of placental transfer of COVID-19 antibodies during birth, especially among those who displayed seropositivity at the study's commencement. This study underscores that maternal antibody levels wield a substantial influence over the effectiveness of COVID-19 antibody transfer through the placenta during pregnancy. In essence, this investigation contributes vital evidence regarding the epidemiological burden of COVID-19 in the context of pregnancy. By pairing these findings with economic and societal burden modeling, our study offers the means to estimate the disease's impact over both the short and long terms. Moreover, this wealth of information aids in the strategic allocation of resources, supports the design and implementation of monitoring and evaluation protocols for disease control policies targeted at the pregnant population, and ultimately facilitates effective planning for the challenges posed by COVID-19.
